# Denial of Reward in the Neonate Shapes Sociability and Serotonergic Activity in the Adult Rat

**DOI:** 10.1371/journal.pone.0033793

**Published:** 2012-03-27

**Authors:** Anastasia Diamantopoulou, Androniki Raftogianni, Antonios Stamatakis, Filaretos Alikaridis, Melly S. Oitzl, Fotini Stylianopoulou

**Affiliations:** 1 Laboratory of Biology-Biochemistry, School of Health Sciences, University of Athens, Athens, Greece; 2 Department of Biological Chemistry, School of Medicine, University of Athens, Athens, Greece; 3 Division of Medical Pharmacology, Leiden University, Leiden, The Netherlands; University of Houston, United States of America

## Abstract

**Background:**

Manipulations of the early environment are linked to long-lasting alterations of emotionality and social capabilities. Denial of rewarding mother-pup interactions in early life of rats could serve as model for child neglect. Negative consequences for social competence in later life, accompanied by changes in the serotonergic system would be expected. In contrast, rewarding mother-pup contact should promote adequate social abilities.

**Methodology/Principal Findings:**

Male Wistar rats trained in a T-maze during postnatal days 10–13 under denial (DER) or permission (RER) of maternal contact were tested for play behavior in adolescence and for coping with defeat in adulthood. We estimated serotonin (5-HT) levels in the brain under basal conditions and following defeat, as well as serotonin receptor 1A (5-HT1A) and serotonin transporter (SERT) expression. DER rats exhibited increased aggressive-like play behavior in adolescence (i.e. increased nape attacks, p<0.0001) and selected a proactive coping style during defeat in adulthood (higher sum of proactive behaviors: number of attacks, flights, rearings and defensive upright posture; p = 0.011, p<0.05 vs RER, non-handled-NH). In adulthood, they had lower 5-HT levels in both the prefrontal cortex (p<0.05 vs RER) and the amygdala (p<0.05 vs NH), increased 5-HT levels following defeat (PFC p<0.0001) and decreased serotonin turnover (amygdala p = 0.008). The number of 5-HT1A immunopositive cells in the CA1 hippocampal area was increased (p<0.05 DER, vs RER, NH); SERT levels in the amygdala were elevated (p<0.05 vs RER, NH), but were lower in the prefrontal cortex (p<0.05 vs NH).

**Conclusions/Significance:**

Denial of expected maternal reward early in life negatively affects sociability and the serotonergic system in a complex manner. We propose that our animal model could contribute to the identification of the neurobiological correlates of early neglect effects on social behavior and coping with challenges, but also in parallel with the effects of a rewarding early-life environment.

## Introduction

Social interactions between parent and child as well as among peers early in life are key factors for an appropriate brain and behavioral development. The consequences of disruption of these interactions or adversity linked to these early life components have been described extensively in humans as well as in animal models (reviewed by [1]–[4]. We have developed a novel paradigm of early life social challenge in the rat that employs contact of the pup with the mother as either a positive (rewarding) or negative (frustrative) reinforcer in a T-maze, during postnatal days 10–13 [5]. The use of this paradigm makes it possible to study in parallel two different early life experiences: one of mild adversity terminated by a positive (receipt of expected maternal reward) event and one of increased adversity mediated by a continuously imposed negative (denial of expected maternal reward) event. We consider ‘denial of maternal reward’ for the neonatal rat a more analogous situation to child neglect than maternal separation per se, as the pup perceives the presence of the mother but is denied contact with her.

The serotonergic system holds a key function in controlling aggression and impulsivity as well as coping styles in response to stimuli threatening an organism's homeostasis. Furthermore, it has a prominent role in embryonic and early postnatal brain development modulating construction and plasticity of new born brain circuits [6]–[10]. Key players of the serotonergic system like the serotonin transporter (SERT), regulating serotonin (5-HT) availability at the synapse, as well as serotonin receptors, like 5-HT1A are shown to be affected by early life experiences. [11]–[16].

It has been well documented that allelic variations in the human SERT gene are associated with emotional and cognitive traits and increased risk for depression, anxiety disorders, hostility and aggression in humans, primates and rodents [17]–[27]. Furthermore, it has been shown that the genotype at the SERT gene locus interacts with early life experiences determining vulnerability to emotional disorders [28], [29]. The role of 5-HT1A receptors in affective disorders and the mechanism of action of antidepressants is well known (reviewed by [30]. In addition, a function of 5-HT1A in the regulation of fear, aggression and impulsivity has also been demonstrated [31]. A link between SERT function and adaptive 5-HT1A alterations has been reported in depressed humans [32] and in SERT knockout mice [33].

The interplay between SERT, 5-HT1A and 5-HT neurotransmission and their sensitivity to adverse early life events in relation to the development of adaptive [34] or abnormal forms of aggression [35], [36] as well as coping styles has been well documented [37], [38]. Thus the study of serotonergic system responses in adulthood on a background of an early life challenge appears crucial. We will use our paradigm of rewarding or denying contact with the mother to investigate the long-term impact on social behaviour and markers of the serotonin system. In adolescence, social play-fighting will be observed and the same rats, when adults, will be tested in their roles as “intruders” in a social defeat paradigm. Immediately thereafter, the serotonergic markers i.e. 5-HT and 5-hydroxyindoleacetic acid (5-HIAA), SERT and 5-HT1A, will be determined, in several brain regions of the adult male rat. In a separate group of rats, serotonergic markers will be measured under basal conditions. We expect that rats who were denied maternal contact during neonatal life will express deficiency in social copying styles along with alterations in the serotonergic system.

## Results

### Denial of maternal reward is linked to a more aggressive-like play in adolescence (Figure 1)

**Figure 1:**
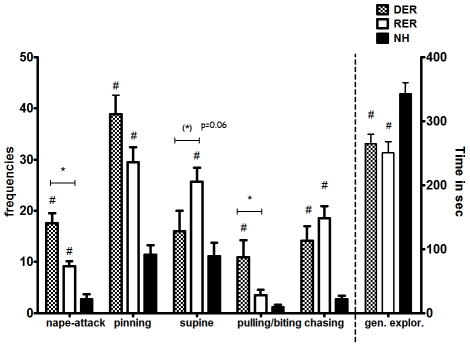
Effect of different early life experience (DER vs RER) in comparison to no experience (NH) during the neonatal period on play-fighting behavior in adolescence. Data in the left part of the graph are means ± S.E.M. of number of nape attacks, pinning and supine behaviors and on the right means ± S.E.M. of duration of general exploration during the 10 min of interaction. #, p<0.05, DER,RER≠NH; *, p<0.05, RER≠DER, post-hoc, one-way ANOVA.

Most of the parameters of play-fighting in adolescence revealed significant differences between the groups: nape attacks (F_(2,24)_ = 25.307, p<0.0001), pinning (F_(2,24)_ = 20.960, p<0.0001), pulling/biting (F_(2,24)_ = 6.897, p = 0.005), chasing (F_(2,24)_ = 12.743 , p<0.0001), supine (F_(2,24)_ = 4.151, p = 0.030), Rats trained in the T-maze as neonates exhibit enhanced interaction with a peer when tested in the social play set up. Adolescent rats trained either under reward or its denial showed increased frequencies for nape attacks, pinning, pulling/biting and chasing the play partner when compared to naïve rats that underwent no training in the neonatal experience (*post-hoc* comparisons using Tukey's test, p<0.05). In contrast, animals with no neonatal experience (NH) spent significantly more time in general exploration of the test box, rather than interacting with peers (F_(2,24)_ = 7.960, p = 0.002, p<0.05 NH vs RER, DER). RER and DER rats differed from each other in their effects on the type of play behavior: DER rats were engaged in more aggressive-like play, shown by significant enhanced nape-attacks (*post-hoc* p = 0.002) and pulling/biting (*post-hoc* p = 0.044), whereas RER rats had a tendency for increased frequencies for supine behavior (p = 0.06).

### Receiving reward during the neonatal period is associated with a reactive coping style during defeat (resident-intruder test) in adulthood whereas denial of reward is associated with a proactive coping (Figure 2)

**Figure 2:**
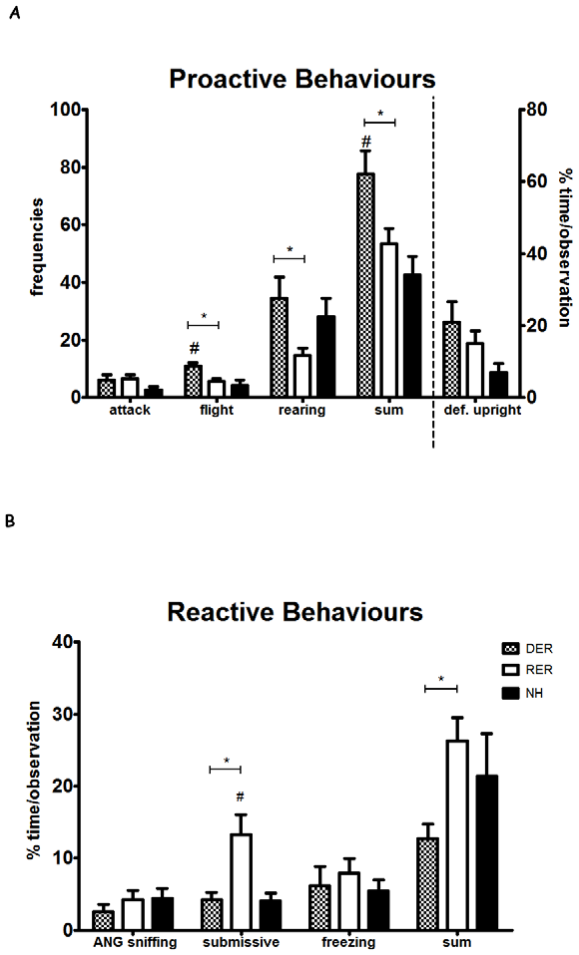
Effect of different early life experiences on proactive-aggressive (A) and on reactive (passive-submissive) (B) behaviors during the defeat phase (10 min) of the resident-intruder test in adulthood. A. Bars on the left part of the graph depict means ± S.E.M. of the number of attacks, flight escapes, rearings during defeat, as well as means ± S.E.M. of total number of the sum of proactive behaviors. Bars on the right part of the graph (separated by a dashed line) represent means ± S.E.M. of duration of upright defensive behavior. B. Bars on the graph depict means ± S.E.M. of duration of anogenital sniffing, full submission and freezing during defeat, as well as means ± S.E.M. of total duration of the sum of submissive behaviors. #, p<0.05, DER, RER≠NH; *, p<0.05, RER≠DER, post-hoc, one-way ANOVA.

To assess the coping strategies of RER, DER and NH rats during social defeat a number of reactive (passive-submissive) and proactive behaviors were scored. When compared to the other two groups (i.e. RER and NH), DER rats showed increased frequencies of proactive behaviors, like flight (F_(2,27)_ = 6.298, p = 0.006, p<0.05 vs RER, NH) and rearing (F_(2,27)_ = 3.675, p = 0.040, p<0.05 vs RER), as well as a higher sum of proactive behaviors (number of attacks, flights, rearings and defensive upright posture; F_(2,27)_ = 5.490, p = 0.011, p<0.05 vs RER, NH). RER rats exhibited increased duration of fully submissive posture upon defeat (F_(2,25)_ = 5.029, p = 0.015, p<0.05 vs DER, NH) and increased percentage of time engaged in reactive/submissive behaviors (F_(2,26)_ = 4.457, p = 0.023, p<0.05 vs DER).

### Rats denied maternal reward as neonates show lower prefrontal and amygdala serotonin levels in adulthood (Figure 3)

**Figure 3:**
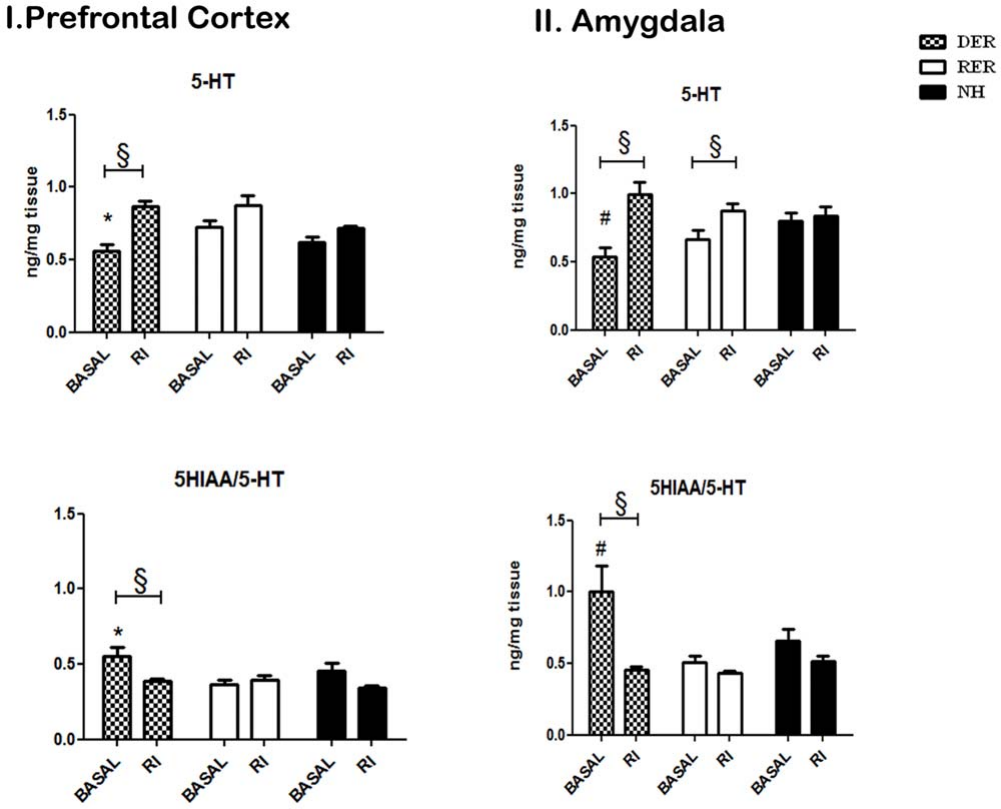
Effect of different early life experiences on basal and stress induced (resident-intruder test) levels of serotonin (5HT) and the ratio, 5-hydroxyindoleacetic acid (5-HIAA)/5-HT, as a measure of serotonin turnover in the prefrontal cortex (A) and amygdala (B). Bars represent means ± S.E.M of amount of monoamines (ng) per mg of tissue. Post-hoc tests on two-way ANOVA, #, p<0.05 DER, RER≠NH at basal condition; *, p<0.05, RER≠DER, post-hoc, §, p<0.05, Basal≠Resident-Intruder, one-way ANOVA.

Under basal conditions, DER rats had lower prefrontal serotonin levels (F_(2,27)_ = 4.122, p = 0.028, p<0.05 vs RER) and increased serotonin turnover (F_(2,28)_ = 3.921, p = 0.032, p<0.05 vs RER) in the prefrontal cortex (PFC). Amygdala (AMY) serotonin levels were lowest in DER rats (F_(2,28)_ = 4.113, p = 0.028, p<0.05 vs NH). Serotonin turnover was increased (F_(2,27)_ = 4.388, p = 0.023, p<0.05 vs RER) in DER rats in the amygdala. Serotonin levels or turnover in hippocampi of DER, RER and NH rats were comparable.

### Social defeat stress affects serotonin levels and turnover only in the brains of DER rats (Figure 3)

Serotonin levels in the prefrontal cortex changed following social defeat in a group-dependent manner (condition-basal vs R.I.-effect F_(1,58)_ = 24.888, p<0.0001, and group x condition interaction F_(2,58)_ = 3.687, p = 0.032). There was a strong tendency for increased levels of this neurotransmitter following defeat in DER and RER animals in comparison to NH (p = 0.051). Social defeat increased serotonin levels in the prefrontal cortex of DER rats (condition effect F_(1,19)_ = 28.578, p<0.0001) but not in RER and NH rats. Similarly, serotonin turnover in prefrontal cortex changed following social defeat in a group-dependent manner (condition-basal vs R.I.-effect F_(1,55)_ = 6.653, p = 0.013, and group x condition interaction F_(2,55)_ = 3.835, p<0.028). Social defeat decreased serotonin turnover in the prefrontal cortex of DER rats (condition effect F_(1,18)_ = 6.554, p<0.020) but not in RER and NH rats.

Serotonin levels in amygdala changed following social defeat in a group-dependent manner (condition-basal vs R.I.-effect F_(1,57)_ = 16.697, p<0.0001, and group x condition interaction F_(2,57)_ = 4.324, p = 0.018). Social defeat increased serotonin levels in amygdala of DER rats (F_(1,19)_ = 15.116, p = 0.001) and RER rats (F_(1,18)_ = 5.770, p = 0.028) but not in NH rats. Serotonin levels did not differ between groups following social defeat stress. Serotonin turnover was affected by social defeat (condition-basal vs R.I.-effect F_(1,53)_ = 10.936, p = 0.002) in a group dependent manner (group x condition interaction F_(2,53)_ = 3.661, p = 0.033). Social defeat decreased serotonin turnover in amygdala of DER rats (F_(1,19)_ = 8.867, p = 0.008), but not in RER and NH rats.

Serotonin levels and turnover in the hippocampus were not affected by the social defeat stress in any of the animal groups.

### Social defeat results in increased corticosterone levels: different early life experiences are not reflected in the defeat stress-induced corticosterone levels

Corticosterone levels were significantly elevated in all three groups after the defeat experience, when compared to the basal levels (repeated measures main effect F(1,26) = 326,783; p = 0.0001). However, there were no differences among the three groups. Corticosterone values following defeat and the percent increase compared to the basal levels are presented in *Table 1*.

**Table 1:** Corticosterone levels following defeat stress.

Groups	DER	RER	NH
Corticosterone after defeat (Means ± S.E.M)	858.30±63.88	820.88±66.73	992.27±57.57
% increase compared to basal	750%	850%	980%

### 5-HT1A receptor levels are affected in the adult brain as a result of an early life history of denied maternal reward (Figure 4)

**Figure 4:**
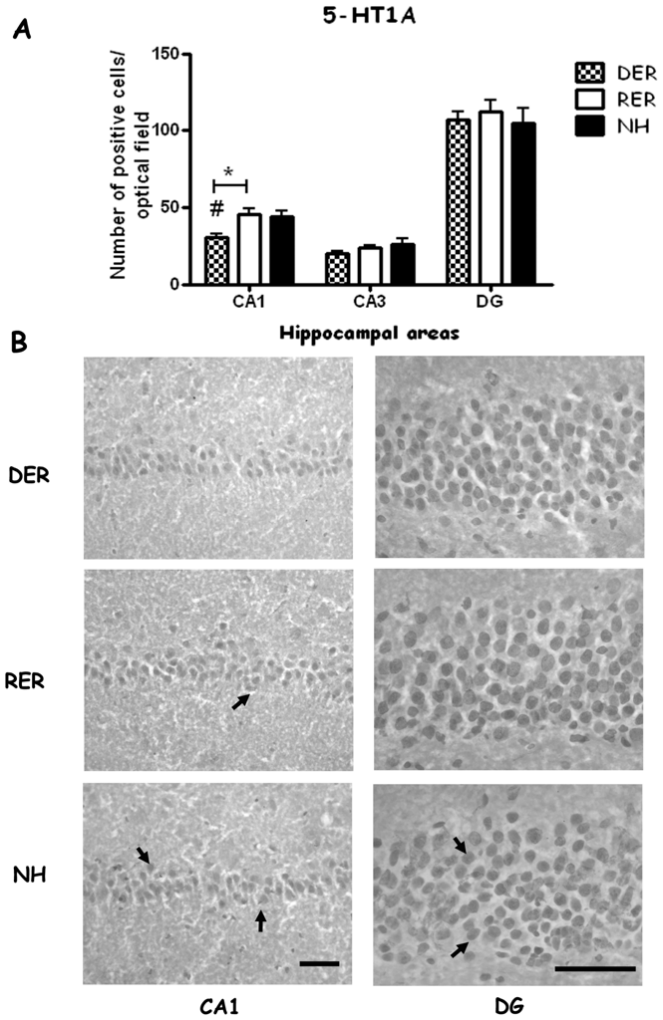
Effect of different early life experiences on 5HT1A receptor protein levels in the hippocampus of adult rats, as depicted by immunohistochemistry. A. Bars represent means ± SEM of immunopositive cells (average of 3 optical fields per area in each sample, n = 6 samples per group). #, p<0.05, DER, RER≠NH; *, p<0.05, RER≠DER, post-hoc, one-way ANOVA. B. Photomicrographs depicting results of immunohistochemistry. Arrows point to immunopositive cells representative of those included in the counting. Scale bar: 50 µM.

The early life experience of denial of an expected reward (DER) was linked to a decrease in the number of 5-HT1A immunopositive cells in the CA1 hippocampal area of adult rats, naïve to any adolescent or adult experience (group effect F(_2,18_) = 6.999, p = 0.007, p<0.05 DER, vs RER, NH). Other brain areas analyzed (CA3, DG hippocampal area, basolateral amygdala, central amygdala, anterior cingulate cortex) were comparable between groups.

### SERT protein levels in the different areas of adult brain are modified in a variable manner based on the nature of the early life experience (Figure 5)

**Figure 5:**
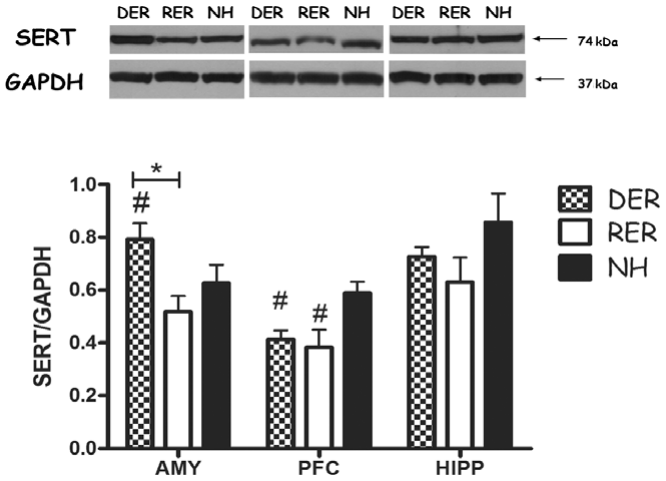
Effect of different early life experiences on serotonin transporter protein levels in the amygdala (AMY), prefrontal cortex (PFC) and hippocampus (HIPP) of adult rats subjected to the resident-intruder test, as revealed by western blot analysis. Bars represent means ± SEM of the optical density (OD) of receptors' band divided by that of the respective GAPDH band in each sample (n = 9 samples per group) #, p<0.05, DER, RER≠NH; *, p<0.05, RER≠DER, post-hoc, one-way ANOVA.

The neonatal experience of denied reward increased protein levels of SERT in the amygdala, when compared to the rewarding experience or non handling (group effect F(_2,24_) = 6.260, p = 0.007, p<0.05 vs RER, NH). In the prefrontal cortex, DER and RER rats had decreased protein levels of SERT (group effect F(_2,25_) = 4.761, p = 0.019, p<0.05 vs NH). SERT protein levels in the hippocampus were comparable between groups.

## Discussion

Rewarding the performance of the rat pup by allowing access to the mother during training on postnatal days 10 to 13 resulted in a less aggressive, although active play during adolescence and reactive coping in a social challenge in adulthood, when compared to control (no handling) rats. Changes in the serotonergic system were not profound. However, denial of maternal reward during training early in life leads to a more aggressive-like play in adolescence and a proactive coping style in adulthood, as well as, reduced serotonin levels in amygdala and prefrontal cortex, increased SERT in amygdala but reduced in prefrontal cortex, and decreased 5-HT1A receptor levels in the CA1 area of the hippocampus. We conclude that denial of maternal contact as a form of early life adversity programs the serotonergic system in its major components and controls certain aspects of social behaviors towards a more proactive/aggressive-like repertoire.

Social play in adolescence apart from serving a primary function, based on its rewarding properties [39], can be considered as necessary for normal development of adult social behavior and social adaptive capacities [39]–[42]. In line with other studies that have imposed early life adversity (i.e. maternal separation, early weaning, post-weaning social isolation) [43], [44]{Veenema, 2009 #63}, we also showed that denial of maternal contact, which can be perceived as frustration, leads to increased nape attacks and pinning during social play. Engagement of rats in a more aggressive-like play in adolescence may be indicative of abnormal social behaviors and could be associated with inadequate affiliative behaviors in adulthood. Such rough play related to early life adversity might bear similarities to aggressive behaviors in maltreated and neglected children [45], serving as a preclinical model to address this issue, given the notion that affiliative behaviors depend on the same brain areas (i.e. amygdala and cingulated cortex) in both rodents and primates/human [46]–[49].

Another issue that has emerged from our results is the reduced social interaction exhibited by the non-handled (NH) control group in comparison to the two early life manipulated groups, during social play. Animals of the NH group spend more time in non-social behaviors (general exploration) than social behaviors compared to the DER and RER animals. This might indicate that no handling or challenge at all during early life leads to an altered phenotype with symptoms of social withdrawal, at least during play-fighting in adolescence.

Early negative social experiences modulate the development of aggressive behaviors. Maternal separation has been reported to promote pro-aggressive behaviors in juvenile mice exposed to the social interaction test [50], enhance aggressive-like play [43] and adult intermale aggression [35]. Furthermore, post-weaning social isolation of male rats increased violent aggression towards intruders [51] and reduced submissive behavior towards residents [42]. Our experiments were not designed to show effects on offensive aggression in the rat, the equivalent of controlled-proactive-instrumental-predatory aggression in humans [52], since the setup was such as to allow display of defensive aggression by rats that were attacked by a dominant (resident) conspecific. This latter form of aggression may be reminiscent of impulsive-reactive-hostile-affective aggression observed in humans [53]. Numerous studies have demonstrated that it is the impulsive form of aggression that is mostly enhanced by early life stress [54]–[58] and appears to be maladaptive. For example, rhesus monkeys in the wild characterized by excessive impulsive and aggressive behaviors were more likely to hold low social dominance (males) and to express abusive maternal behaviors (females) [59], [60]. High aggression in humans as a result of early childhood trauma correlates with increased suicide attempts [61]. Our data show that a negative early life experience in the form of denial of maternal reward is indeed linked to decrease of submissive-reactive behaviors and increase of proactive behaviors, nevertheless fails to increase defensive aggression per se. However, the proactive coping style is characterized by increased levels of offensive aggression, impulsive decision-making and a rather decreased behavioral flexibility (increased rigidity) [62]–[64]. In this respect, rats denied the expected maternal reward as neonates show a more proactive coping style upon defeat and increased aggression during play and might appear less adaptive to environmental changes.

Receipt of maternal reward early in life was linked to increased interest for non aggressive social play in adolescence and an appropriate adaptive (submissive) behavior upon defeat by a dominant resident conspecific in adulthood. We propose that their reactive coping style will render the RER rats more adaptable under variable and unpredictable environmental conditions and is likely to make them more resilient to stress load accumulating by these conditions. Coping styles have been defined as alternative response patterns in reaction to a stressor and, within this framework, individual variation in aggression could possibly be an expression of actively coping with environmental challenges in general [65]. In relevance to our results, it has been previously shown [65] that when low-aggressive intruders are placed as intruders within the home-territory of a trained highly-aggressive resident (i.e., the defensive aggression test), they quickly adopt a submissive freezing-like response upon a resident attack, as in the case of the RER animals. On the other hand, aggressive animals tend to proactively flee from the situation, as in the case of our DER rats.

The proactive coping style of the DER animals is coupled to reduced serotonin levels in their amygdala and prefrontal cortex. Substantial evidence indicates that serotonin is causally involved in both aggression and behavioral flexibility; high levels of aggressive and impulsive behavior are associated with low levels of brain serotonin in the PFC [18], [38], [66]–[69].

Concerning SERT brain levels, our results corroborate with other studies examining the effects of early neonatal manipulations on SERT levels in the adult rat brain. Specifically, increased levels of SERT in the amygdala were found in a brief (15 min) maternal separation group (handling) [13].

Levels of post-synaptic 5-HT1A receptor seem to be under developmental control, since the neonatal experience of denied maternal reward is followed by decrease in 5-HT1A receptor levels in the CA1 area of the hippocampus. The CA1 hippocampal area appears to be particularly sensitive to early life manipulations, since analogous results to ours have been reported by Meerlo et al. They employed a different early life intervention i.e. corticosterone administration during late gestation, and showed that offspring had decreased 5-HT1A receptor binding in the CA1 hippocampal area in adulthood [70]. We hypothesize that experiencing denial of maternal contact can be considered stressful enough to elicit a corticosterone response. Such an increase of corticosterone levels in the neonates experiencing denial of maternal reward might similarly program a downregulation of 5-HT1A receptor levels in the adult hippocampal CA1 region. Furthermore, it has been showed that early maternal separation has been shown to affect 5-HT1A receptor densities in hippocampal areas and amygdala in the *Octodon degus*, in amygdalar nuclei of adult rats and mRNA in the CA1 hippocampal area of rat pups [11]–[14]. Interestingly the decrease in 5-HT1A was observed in the absence of DER-induced changes in the levels of serotonin or its metabolites in the hippocampus. A similar dissociation has been reported using the neonatal handling paradigm, which leads in males to increased 5HT1A receptor levels in the hippocampus, without affecting serotonin [71], [72]. The DER-induced decrease in 5HT1A seems to be a specific effect localized exclusively to the CA1 area, representing a distinct mechanism modulating synaptic function, without affecting hippocampal serotonergic neurotransmission globally.

Reduced serotonin, increased SERT levels in the amygdala and reduced 5-HT1A receptor immunoreactivity in the CA1 hippocampal area found in the DER animals support a reduced serotonergic activity in the brain that could be linked to their proactive coping and increased aggressive-like behavior. Furthermore, it has been shown that the expression of abnormal aggression patterns can be triggered by reduction of serotonergic neurotransmission [49], [69], [73]. SERT knockout rats exhibit enhanced PFC serotonin and reduced aggression as measured in the resident-intruder paradigm, as well as improved inhibitory control [22], [74]. In humans, childhood emotional neglect was associated with lower levels of the 5-HT metabolite 5-hydroxyindoleacetic acid (5-HIAA) in adults [75]. Decreased 5-HT activity in certain brain regions may be a crucial predisposing mechanism involved in the development of excessive aggressive behaviors in maternally deprived monkeys [59], [76], [77]. Therefore, the overall decreased serotonergic activity in the DER animals could also be associated to their negative early life history. In more detail, whereas decreased SERT PFC levels might serve as a compensatory mechanism to the decrease in serotonin in an attempt to restore serotonergic activity in that region, in the amygdala increased SERT levels accompanied by reduced serotonin leading to a decreased overall serotonergic activity in this brain region might indicate a less appropriate social decoding of fear-defeat signals elicited by the resident. In relation to our data, there is evidence from studies both in rodents and humans that amygdala plays a key role in decoding aggression related emotional stimuli [49], [78]–[80].

Serotonin (5-HT) levels have been shown to increase following a number of acute stressors (e.g. footshock, restraint, exposure of mice to rats) [81], [82] as well as social defeat [83]. We showed elevation of 5-HT levels in the prefrontal cortex and amygdala of adult rats following social defeat only on the background of a negative early life experience (DER). This result might indicate that a demanding early life social environment increases the responsivity of relevant brain systems to an analogous challenge in the long term.

The DER experience has elements of adversity, although of a milder nature than most maternal deprivation paradigms. Interestingly both DER and maternal deprivation have similar consequences on behavior, both increasing aggressive-like play in adolescence and resulting in a proactive coping strategy upon defeat in adulthood. However the underlying neurobiological effects induced by the two early experiences are different. More specifically maternal separation results in increased basal and stressed-induced corticosterone levels and increased anxiety [84]–[87], while the DER experience does not affect corticosterone levels under basal conditions (data not shown), or following defeat, or anxiety as measured in the elevated plus maze (data not shown). As far as the serotonergic system is concerned, both early experiences – the DER and maternal deprivation – result in increased SERT levels in the amygdala . However no DER-induced changes in amygdalar 5-HT1A receptor levels were observed in our study, in contrast to the reported maternal deprivation-induced increase in 5-HT1A in this brain area, compared to non-handled animals [13].

Our above findings could suggest that an aversive experience in a critical developmental period induces plasticity and rewiring of associated brain systems leading to a “differential susceptibility” for challenges in later life. The basis of this hypothesis relies on recent developments in psychiatric research which propose that individuals most susceptible to adversity because of their genetic makeup have been shown to be simultaneously most likely to benefit from supporting or enriching experiences or just in the absence of adversity [88]–[90]. In this respect, “enrichment” early in life through mild adversity might trigger plasticity in multiple brain systems and differential regulation of gene expression. This effect, along with future life events will define an individual's vulnerability for psychopathology or adaptive plasticity.

In conclusion, we have shown that denial of expected maternal reward early in life affects sociability, coping style and expression of key elements of the serotonergic system controlling these behaviors in a complex manner. Our animal model of maternal reward and its denial is of high translational value for child neglect: maternal presence and short separation, as is mostly the case in the human situation, grants it with high ecological validity. We believe that this experimental paradigm could possibly serve as a means of further investigating the neurobiological correlates of early neglect effects on social behavior and coping with challenges, in parallel with the effects of an enriching and rewarding early life.

## Materials and Methods

### Ethics Statement

Experiments were carried out in agreement with recommendation of the European Communities Council Directive of 22 September 2010 (2010/63/EU).

### Animals

A total of 100 rats (Wistar, laboratory colony) derived from 16 litters used as experimental subjects (n = 40 males) and partners for social encounters (n = 40 males), and as residents (n = 10 males co-housed with n = 10 females) in social defeat experiments. Rats were kept under standard conditions (24°C, 12∶12 h light/dark cycle; light on at 5:00 with food and water ad libitum. The day of birth was designated as postnatal day 0 (pnd). Litters were assigned randomly to three groups (1) pups receiving the expected reward – RER; (2) pups denied the expected reward - DER; (3), pups of the control group: non-handled –NH. To maintain stable environmental stimulation for the pups, instead of cleaning the cage, wood chip was added every 4–5 days for all groups. Weaning took place on pnd 22, and 3–4 rats were housed per cage. For each experimental group we used 2–3 males from four different litters. A separate cohort comprising 2 males from 3 different litters of each experimental group (n = 6 per group) was used for immunohistochemistry analysis.

### Neonatal training in the T-maze

We used a custom-made T-maze (for dimensions and details see Panagiotaropoulos et al, 2009), at the end of the right arm of which a small sliding door permitted access to the cage with the mother and litter when pups were trained under continuous reinforcement (receiving expected reward-RER). The door remained closed, preventing entrance in the mother-litter's cage, when pups were trained under continuous frustration (denied expected reward-DER). For control purposes, we placed a cage containing a virgin female rat at the end of the left arm of the T-maze. Pups were trained in the T-maze on postnatal days 10, 11, 12, and 13 (Figure 1). For further details on the procedures see [5], [91].

### Play-fighting at adolescence

Play-fighting was assessed at 5-weeks of age (pnd35), as play-fighting is highest at this age [92]. To stimulate play-fighting [93] rats were housed singly for 4 hrs (DER: n = 11; RER: n = 10; non-handled NH: n = 10) At the beginning of the dark cycle (between 17:00 h and 19:00 h) rats were exposed in their home cage to an unknown age- and weight-matched male Wistar rat (not isolated) for a period of 10 min (adapted from [43]. Behavior was videotaped and scored by using ‘*Registration’* software: durations and frequencies of pinning, nape attack, supine, approaching/following (moving in the direction of the partner), social exploration (grooming or sniffing the body of the partner), and nonsocial behaviors (self-grooming, rearing, and exploration of the cage). The same rats were used as intruders in the social encounter test at adulthood.

### Resident-intruder test at adulthood

‘Resident” rats (6 months old) had been trained to exhibit aggressive behavior. They were housed in a separate room in large cages (79×57×42 cm) with a female to stimulate territorial aggression and trained on a regular basis (daily for at least 2 weeks) by introducing young naïve males. Residents with attack latency shorter than 2 min were selected. At 3 months of age, rats of the three experimental groups were designated as ‘intruders’, which should experience the social defeat stress. Intruder rats were transferred in their home cage to the room of the residents and immediately put into the resident's cage. At the same time, the female partner of the resident was removed. We allowed 10 min for physical resident-intruder contact: attack and defeat (indicated by submissive behavior, with the animals lying motionless on their back), then, the intruder was confined into a wire mesh cage inside the resident's cage for another 30 min. Auditory, olfactory, and visual stimuli of the resident remain for a standard period, which is experienced as threat after the actual defeat and known to be highly stressful [94]. The resident-intruder test took place in the dark period under red light, was video-taped and the behavioral scoring was done using the *Registration* software.

#### Behavioral analysis

To identify the coping styles of the rats, their behaviors were categorized either as passive-submissive (reactive), or as pro-active, as described in Table 2. According to literature, aggressive male rats have a more proactive behavioral response linked to behavioral rigidity, whereas non-aggressive or reactive (submissive) males appear to be more adaptive, responding only when necessary [68], [95], [96]. Therefore, apart from scoring individual behaviors we also calculated the sum of passive-submissive and proactive behaviors (duration and/or frequencies; see Table 2).

**Table 2:** Behaviors during resident-intruder test.

Coping style	Description	Behaviors
Passive-submissive (reactive)	Behaviors with vigilance, anxiety-related, fear-related, or risk assessment components as well as submissive behaviors	Passive genital sniff, genital sniff, freezing,sideways submission, and full submission
Proactive	Confrontational behaviors and behaviors with exploration or escape components	Attack, upright defensive behavior, rearing, and escape

Adapted from Gardner et al, 2005 [97].

### High-performance liquid chromatography –HPLC

Immediately after the end of the social defeat stress rats were decapitated, brains were removed and placed on ice-cold glass plates for dissection. For the estimation of non-stressed baseline conditions, DER, RER and NH rats of the same age were used. The prefrontal cortex, hippocampus and amygdala were dissected from each hemisphere. Areas from the right hemisphere were processed for HPLC analysis, whereas those of the left were used for Western blotting. Brain structures were weighed and homogenized in 0.2 N perchloric acid (HClO_4_) containing 1O^−4^ M ascorbic acid. After centrifugation (12 000 r.p.m., 15 min, 4°C) the supernatant was stored at −80°C until assay. Concentrations of serotonin (5-HT) and its metabolite 5-hydroxyindoleacetic acid (5-HIAA) were measured by HPLC using electrochemical detection, as previously described [72], [98].

### Western Blotting

Brain areas were homogenized by sonication in lysis buffer [20 mM Tris HCl pH 7.6, 137 mM NaCl, 48 mM NaF, 2 mM Na_3_VO_4,_ 1% SDS, and 10% glycerol, 1∶250 Protease Inhibitor Cocktail (Sigma)] and centrifuged at 14,000 rpm for 20 min at 4°C. 65 µg of total protein was loaded for each brain area to 4–12% NuPAGE® Bis-Tris precast polyacrylamide gels (Invitrogen, USA). Proteins were then transferred to 0.45 µm nitrocellulose membranes (Whatman) and incubated for 2 h at room temperature with 5% nonfat dried milk in to reduce nonspecific binding. The upper part of the membranes was incubated with Anti-Serotonin Transporter rabbit polyclonal antibody, 1∶2000 (Millipore) and the lower part of the membranes with anti-GAPDH mouse monoclonal antibody, 1∶1000 (Millipore), overnight at 4°C. Following 2 h incubation with an HRP-conjugated goat anti-rabbit (Santa Cruz Biotechnology Inc., 1∶10000) or rabbit anti-mouse (Santa Cruz Biotechnology Inc., 1∶5000) secondary antibody, respectively at room temperature and the signal was visualized on autoradiographic films (Kodak XAR) by chemiluminescence (Amersham, UK). Optical density of each band was determined using the Image J Software (Image J Gel Analysis method) where divided by the respective GAPDH band reading (relative optical density ratio).

### Immunohistochemistry and quantification of immunopositive cells

Rats were deeply anesthetized, decapitated and brains were isolated and frozen in −40°C isopentane. Twenty micrometer sections were cut on a cryostat (Leica CM1900, Nussloch, Germany) at −17°C, collected on silane-coated slides and stored at −80°C until further processing. Imminohistochemistry for 5-HT1A receptors was performed as previously described [71]. Tissues from all groups (DER, RER, NH) were processed concurrently. Brain areas were defined according to Paxinos and Watson [99]. Cell counting in the prefrontal cortex (infralimbic, anterior cingulate), the amygdala (BLA, CeA) and the hippocampus [CA1, CA3, dentate gyrus (DG)] was performed “blindly” by two independent investigators using “Image Pro Plus” (Media Cybernetics, USA) in three sections of each brain area of each rat. A threshold for non-specific background staining was set to allow counting of specifically immunostained cells.

### Radioimmunoassay (RIA) for corticosterone (CORT)

Plasma samples were collected from tail vein blood, for determination of plasma corticosterone (CORT) under basal conditions (on day 80 of age) and from trunk blood after the end of social defeat. Samples were collected in ice-chilled EDTA coated microcapillary tubes (Sarstedt), which were then centrifuged at 3000 rpm for 10 min to collect plasma. All plasma samples were stored at −20°C until radioimmunoassay (RIA). Plasma corticosterone concentrations were measured using commercially available radio immunoassay (RIA) kits containing ^125^Iodine labelled corticosterone (variance: intra-assay 6.9%, inter-assay 7.3%, respectively (ICN Biomedicals Inc., CA). Corticosterone concentrations were determined in duplicate against an extended standard curve (0, 3, 6.25, 12.5, 25, 50, 100, 250, 500, 1000 ng corticosterone/ml). Vials were counted for 2 min in a gamma-scintillation counter (Packard Minaxi Gamma counter, Series 5000).

### Statistical analysis

Neurochemical data from HPLC were analyzed by two-way analysis of variance (ANOVA) with the group of rats (DER, RER, NH) and the experimental condition (basal, resident-intruder stress) as the independent factors. Neurochemical data from Westerm blots and immunohistochemistry, and behavioural data from play-fighting and social defeat were analyzed using one-way ANOVA with the group of rats (DER, RER, NH) as the independent factor. When main effects or interactions between the independent factors were detected, Tukey's post hoc tests were performed. Data were deemed significant when p<0.05. Results are expressed as mean ± standard error of the mean (SEM).
